# Lung cancer screening: what do long‐term smokers know and believe?

**DOI:** 10.1111/hex.12433

**Published:** 2015-12-23

**Authors:** Lisa Carter‐Harris, DuyKhanh Pham Ceppa, Nasser Hanna, Susan M. Rawl

**Affiliations:** ^1^ Indiana University School of NursingIndianapolisINUSA; ^2^ Indiana University School of MedicineIndianapolisINUSA; ^3^ Indiana University Simon Cancer CenterIndianapolisINUSA

**Keywords:** health behaviours, health beliefs, lung cancer screening, primary care providers, smokers

## Abstract

**Objective:**

To explore knowledge and beliefs of long‐term smokers about lung cancer, associated risk factors and lung cancer screening.

**Design:**

Qualitative study theoretically framed by the expanded Health Belief Model based on four focus group discussions. Content analysis was performed to identify themes of knowledge and beliefs about lung cancer, associated risk factors and lung cancer screening among long‐term smokers' who had and had not been screened for lung cancer.

**Methods:**

Twenty‐six long‐term smokers were recruited; two groups (*n* = 9; *n* = 3) had recently been screened and two groups (*n* = 7; *n* = 7) had never been screened.

**Results:**

While most agreed lung cancer is deadly, confusion or inaccurate information exists regarding the causes and associated risk factors. Knowledge related to lung cancer screening and how it is performed was low; awareness of long‐term smoking's association with lung cancer risk remains suboptimal. Perceived benefits of screening identified include: (i) finding lung cancer early; (ii) giving peace of mind; and (iii) motivation to quit smoking. Perceived barriers to screening identified include: (i) inconvenience; (ii) distrust; and (iii) stigma.

**Conclusions:**

Perceived barriers to lung cancer screening, such as distrust and stigma, must be addressed as lung cancer screening becomes more widely implemented. Heightened levels of health‐care system distrust may impact successful implementation of screening programmes. Perceived smoking‐related stigma may lead to low levels of patient engagement with medical care and decreased cancer screening participation. It is also important to determine modifiable targets for intervention to enhance the shared decision‐making process between health‐care providers and their high‐risk patients.

Lung cancer is the leading cause of cancer‐related death in the United States regardless of gender or ethnicity.^1^ More than 50% of these patients are diagnosed with advanced, incurable disease^2^; individuals with Stage III and IV lung cancer have 5‐year relative survival rates of 5% and 1%, respectively.^1^, ^3^ Tobacco smoking is the number one risk factor for lung cancer and has been linked to 90% of all lung cancer cases. Long‐term smokers age 55 or older who have a minimum of a 30 pack‐year tobacco smoking history and currently smoke, or have quit within the past 15 years, are at greatest risk for the development of lung cancer. Pack‐year is defined as number of packs of cigarettes smoked per day multiplied by number of years smoked. Current lung cancer screening guidelines are focused on long‐term smokers.^4^ The term ‘long‐term smoker’ is frequently defined as individuals with a 30 pack‐year or more tobacco smoking history in lung cancer screening guidelines and is defined as such in this paper.^4^, ^5^, ^6^ This study was undertaken to explore the knowledge and beliefs of long‐term smokers about lung cancer, associated risk factors and lung cancer screening.

## Lung cancer screening

Lung cancer screening with low‐dose computed tomography (LDCT) in long‐term smokers has been shown to decrease relative lung cancer‐related mortality by 20%.^7^ In response to empiric findings, the United States Preventive Services Task Force (USPSTF) has issued lung cancer screening guidelines recommending annual LDCT for long‐term smokers.^5^ The USPSTF's Grade B recommendation reflects their conclusion that available evidence was sufficient, with high certainty, that annual LDCT will yield moderate to substantial benefits for this high‐risk group. Further, in the United States (US), the Centers for Medicare and Medicaid Services (CMS) approved coverage of LDCT for its high‐risk members in February 2015.^8^

As lung cancer screening becomes widely implemented, participation is likely to be influenced by many factors, including individual, provider and health‐care system‐related variables.^3^, ^9^, ^10^, ^11^, ^12^ With the recent release of screening guidelines in the USA, efforts to increase provider and health‐care system awareness are underway.^6^, ^13^ The American Cancer Society published a continuing medical education programme to educate providers on: (i) the new guidelines; (ii) results of lung cancer screening clinical trials; (iii) benefits versus risks of LDCT; and (iv) how to identify patients for whom lung cancer screening is appropriate.^4^ Many health‐care systems have incorporated education and training about these new guidelines and are establishing formal lung cancer screening programmes. There are currently 341 lung cancer screening (LCS) centres of excellence as designated by the Lung Cancer Alliance^14^ as well as numerous others across the United States without this designation.

Although enhancing provider awareness is essential, it is also critical to understand factors that influence screening participation among people for whom screening is recommended. Further, understanding individual health beliefs about screening among long‐term smokers will help future efforts to facilitate shared decision making about lung cancer screening participation, which is a requirement of CMS coverage, and essential for all screening‐eligible patients regardless of health‐care coverage.^8^

## Health belief model

The expanded Health Belief Model has been used extensively to understand cancer prevention and control behaviours, including screening.^15^, ^16^, ^17^, ^18^, ^19^ For many cancers, researchers have documented the influence of perceived risk of the cancer as well as perceived benefits of, perceived barriers to, and self‐efficacy for screening.^16^, ^17^, ^18^ As the model predicts, individuals will participate in cancer screening if they believe: (i) they are at risk for a cancer (*perceived risk*); (ii) screening will reduce the consequences through early detection (*perceived benefits*); (iii) benefits to participating in screening outweigh the *perceived barriers*; and (iv) they can accomplish the tasks necessary to complete the screening process (*self‐efficacy*).^20^, ^21^ For behaviour change to occur in long‐term smokers related to lung cancer screening, individuals must believe they are at risk for lung cancer and that getting screened will benefit them. The purpose of this study was to explore long‐term smokers' perceptions of lung cancer, lung cancer risk factors and lung cancer screening. Therefore, the research questions were: (i) What do long‐term smokers know and believe about lung cancer and associated risk factors? and (ii) What do long‐term smokers know and believe about lung cancer screening?

## Methods

A qualitative research approach using focus group discussions was chosen since this method is recommended for collecting data involving perceptions in a defined area of interest in a permissive, non‐threatening environment.^22^ Data from focus group discussions can enhance understanding a particular phenomenon.^23^ The study was approved by the Institutional Review Board at Indiana University.

### Participants and recruitment procedures

Two categories of participants were recruited from the central Indiana area: screened (*n* = 12) and unscreened (*n* = 14) long‐term smokers. We believed it was important to include both groups in the study to achieve heterogeneity in perspectives based on a salient stratifying variable. Twenty‐six long‐term smokers participated in one of four focus group discussions. Two focus groups (*n* = 9; *n* = 3) were comprised of long‐term smokers who had recently participated in lung cancer screening and two focus groups (*n* = 7; *n* = 7) were comprised of long‐term smokers who had never been screened. Inclusion criteria mirrored the USPSTF lung cancer screening guidelines: (i) age 55–80 years; (ii) current or former smoker that has quit within the past 15 years; and (iii) 30 pack‐year tobacco smoking history. Each participant in all four focus groups received a $30 gift card and an informational brochure on lung cancer risk and screening at completion of the focus group discussion.

#### Recruiting screened participants

To identify and recruit participants who had undergone lung cancer screening with LDCT, the research team member who was the medical director of the lung screening clinic at Indiana University Health (author DC) identified individuals who met the inclusion criteria. A letter introducing the study signed by the principal investigator (LCH) and the medical director (DC), as well as a recruitment brochure, were mailed to 41 potential participants. The introductory letter explained that the recipients might be contacted regarding this study and included a local telephone number to call to leave a message if they wished to opt out. A trained research assistant called individuals who did not opt out 1 week after letters were mailed to explain the study, answer questions and invite participation. Of the 41 letters mailed, two persons called to opt out and 17 were successfully contacted by telephone for recruitment. Consistent with focus group methodology to assemble 6–8 participants per focus group,^22^ the remaining 22 potential participants were not contacted because the number needed had been reached. Of the 17 contacted, 14 (82.4%) agreed to participate and 12 (70.6%) attended one of the two focus group discussions for screened participants.

#### Recruiting unscreened participants

To recruit long‐term smokers who had not been screened for lung cancer, community‐based recruitment efforts were employed. Recruitment flyers were placed in high traffic areas (i.e. bus stops, convenience stores and veterans' community halls), and one advertisement that ran for 3 days was placed in the local newspaper. Forty‐seven interested participants called the research office to obtain additional information and were screened for eligibility. Thirty (64%) were eligible; of those, 14 (47%) agreed to participate and attend a focus group discussion; seven attended the third focus group and seven attended the fourth focus group.

### Focus group discussions

After obtaining written informed consent, experienced moderators led the focus group discussions using a semi‐structured moderator's guide derived from a comprehensive review of the literature (see Table 1 for a sample of the questions). Both moderators are doctorally prepared behavioural scientists with experience in health behaviour research related to cancer screening. Discussions were digitally recorded and subsequently transcribed verbatim by a professional transcriptionist. The first author (LCH) compared the transcripts to the audio recordings and made corrections as needed. The transcripts were reviewed independently by two researchers (LCH and SMR).

**Table 1:** Sample items from the semi‐structured moderator's guide

• When I say the word lung cancer, what things come to mind?
• Who do you think gets lung cancer?
• Do you think that lung cancer has symptoms before it is discovered? If so, what are some symptoms of lung cancer?
• How do doctors find lung cancer?
• Do you think that lung cancer can be treated? Cured?
• Do you think that lung cancer can be prevented? If so, how?
• What is cancer screening?
• What is lung cancer screening? How many of you have ever heard of lung cancer screening?
• Do you think that lung cancer can be found early? How is that done?
• Why would you want to find lung cancer early?
• Have you ever heard of a lung scan? Can you describe it?
• Who do you think should have this test? Why?
• Do you think you should have the test? Why? How important do you think it is for you to have this test done?

### Qualitative data analysis

Data were analysed using a standard content analysis process to identify themes in the transcribed text.^24^, ^25^ Neuendorf^24^ defines content analysis as a systematic and objective analysis of message characteristics in a narrative. Content analysis is an appropriate technique when researchers aim to make sense of qualitative data and identify core consistencies and meanings.^25^ Two researchers with expertise in cancer screening research conducted the data analysis of the focus group transcripts. The researchers read all the transcripts in their entirety several times to become familiar with the data. The researchers independently coded the transcripts by providing labels for each relevant text unit, which was any word, phrase, sentence, or story that provided information to address the research questions. The text units included information about participants' knowledge and beliefs about lung cancer, associated risk factors and lung cancer screening. A coding matrix was created using a Microsoft Word table format to display the relevant, identified text units. The 26 participants (12 participants who had been screened and 14 participants who had never been screened for lung cancer) were represented by the rows on the matrix, and the categories exploring participants' knowledge and beliefs about lung cancer, associated risk factors and lung cancer screening were represented by the columns. The text units in the columns were then compared and contrasted and independently grouped into subcategories by the two researchers (LCH and SMR). The researchers then met to discuss themes that emerged from individual coding and to compare the degree of congruence between coding and classifications. Discrepancies were discussed until consensus was reached.

## Results

Participants were a mean age of 66 years (SD 6.3) and had a mean education level of 14.7 years (SD 3.6). Most were Caucasian (76.9%) and retired (76.9%). Almost half (46.2%) were current smokers and the remainder were former smokers. In addition, 9 (34.6%) participants reported a positive family history of lung cancer. See Table 2 for complete sociodemographic characteristics by screening status. While we conducted separate focus groups with screened and unscreened participants expecting to find key differences between groups, the analysis revealed more similarities than differences. The key differences noted were related to perceptions about lung cancer risk and aetiology. Unscreened participants reported lung cancer was mostly caused by environmental insults and occupational exposure, whereas screened participants felt environmental exposure combined with genetic predisposition for lung cancer was a prevailing risk for the development of lung cancer. In the following section, we will discuss findings common in all four groups.

**Table 2:** Sociodemographic characteristics by screening status

	Total (*n* = 26)	Screened (*n* = 12)	Unscreened (*n* = 14)
	*n* (%)	*n* (%)	*n* (%)
Gender			
Female	18 (69)	7 (39)	11 (61)
Male	8 (31)	5 (63)	3 (37)
Race/Ethnicity			
Caucasian	20 (77)	11 (55)	9 (45)
African American	5 (20)	0 (0)	5 (100)
Hispanic	1 (3)	1 (100)	0 (0)
Marital Status			
Married	7 (27)	6 (86)	1 (14)
Divorced	4 (15)	1 (25)	3 (75)
Widowed	4 (15)	1 (25)	3 (75)
Single	11 (43)	4 (36)	7 (64)
Employment			
Retired	20 (77)	8 (40)	12 (60)
Full‐time	5 (19)	4 (80)	1 (20)
Part‐time	1 (4)	0 (0)	1 (100)
Annual Income			
<$20k	7 (27)	1 (14)	6 (86)
$20 001–40 000	8 (31)	5 (63)	3 (37)
$40 001–60 000	8 (31)	4 (50)	4 (50)
$60 001–80 000	1 (4)	0 (0)	1 (100)
More than $80 000	2 (7)	2 (100)	0 (0)
Insurance Status			
Medicare	15 (58)	5 (33)	10 (67)
Medicaid	1 (4)	0 (0)	1 (100)
Private Insurance	8 (31)	7 (88)	1 (12)
No Insurance	2 (7)	0 (0)	2 (100)
Smoking Status			
Current Smoker	12 (46)	3 (25)	9 (75)
Former Smoker	14 (54)	9 (64)	5 (36)
Family History of Lung Cancer			
Yes	9 (35)	4 (44)	5 (56)
No	17 (65)	8 (47)	9 (53)

	Total (*n* = 26)	Screened (*n* = 12)	Unscreened (*n* = 14)
	Mean (SD)	Mean (SD)	Mean (SD)
Age (years)	65.7 (6.3)	66.8 (7.2)	64.8 (5.6)
Education (years)	14.7 (3.6)	14.1 (4.4)	15.2 (3.0)
Pack‐year History	63.6 (31.3)	59.3 (19.8)	67.2 (39.0)

### Long‐term smokers' knowledge and beliefs about lung cancer and associated risk factors

Although knowledge of lung cancer risk factors and causes varied, lung cancer was consistently described as a disease that always leads to death. When asked for details, participants described lung cancer as dying a ‘horrible death’. The thought of lung cancer stimulated images of chemotherapy, smoking, breathing, inactivity, mental strain and fear for participants.

When asked about the causes of lung cancer, most participants focused primarily on environmental and occupational exposures, emph‐asizing tobacco smoking less as a cause of lung cancer. Participants described individuals at risk as those who worked in machine shops and factories, construction, welding and jobs that exposed them to asbestos, benzenes or diesel fumes. Several participants cited environmental exposure related to overseas military deployment as a factor that could increase an individual's risk of getting lung cancer. Tobacco smoking was consistently identified as a risk factor only late in the discussion. Other important perceived causes of lung cancer identified by participants were second‐hand smoke, genetics, and having tuberculosis as a child or young adult. When asked who gets lung cancer, participants identified older individuals and people who have had tuberculosis as most susceptible. Illustrative comments related to occupational exposure included:

My uncle was a plumber for most of his life, and the stuff they put on pipes gets on their skin…the toxins, the benzenes that he was exposed to through the chemicals in plumbing, may have caused his early demise.

I'm a bus driver and all of our busses are inside the garage right now, and of course we're supposed to have ventilation…but the diesel fuel…and all of these other types of fuels that we have…you see the smoke, you see the dust that's accumulated, you go…you breathe in stuff.

Illustrative comments related to environmental exposure, age, genetics and tobacco smoke included the following: ‘There's environmental aspects we don't like to talk about, but they're out there, even here’; ‘There's all kinds of things that we inhale that can be detrimental to our lungs…’; ‘I think that as we get older, we become more vulnerable to some things’; ‘I don't know if I'm susceptible, but I wonder. And yes, I do think there is a genetic component’; and ‘Years and years ago I started smoking when I was very young. I was only 14 and back in those days, we didn't have the knowledge of how bad cigarette smoke was on you’.

During discussions about perceived causes of lung cancer, participants conveyed distrust of the tobacco industry, noting they believed the industry was primarily concerned with profitability and ‘in bed with the government’. Illustrative comments of distrust of both the government and tobacco industry include: ‘Big business, by not smoking, by quitting smoking. Every pack that you don't spend…every time you don't do that, you're keeping money out of big business's pocket’ and

*…*but they sell it. They tax it. They push it. Why is it that they don't start there, that it kills so many people? Every time there's a defect with General Motors or something, there's a recall, and some about people dying, they're having accidents. Well, how come they don't have recalls? [Female interjects, ‘They should recall cigarettes’.] Recall cigarettes, and alcohol, and guns, you know, and they can't do that. They can't get that right because there's so much money, there so much politics, and there's so much everything else.

Overwhelmingly, participants believed lung cancer was preventable. When asked about ways to prevent lung cancer, participants suggested not smoking and protecting one's self from fumes and chemicals in the work place. In addition, a small number of participants noted that individuals are exposed on a daily basis to ‘seen and unseen contaminants’; one noted, ‘it cannot be prevented unless you live in a bowl’.

When asked about the likelihood of lung cancer being cured, participants expressed varying opinions. Some believed lung cancer could be cured by surgery, with statements such as ‘I'd say probably 80/20. Maybe 20%, 80% you're not cured’. Others indicated radiation and chemotherapy could cure lung cancer. Still others said there were so many types of lung cancer that they doubted cure was an option.

### Long‐term smokers' knowledge and beliefs about lung cancer screening

The following section will present findings from the discussion on lung cancer screening awareness, including who should be screened and perceived benefits of and barriers to screening.

#### Awareness of lung cancer screening

When asked about lung cancer screening, most participants were unaware that screening guidelines existed, and some reported not being aware of how screening is performed. Those who were aware expressed confusion about how it was performed. One participant stated, ‘You get the chest X‐ray first, then you get the CAT scan, then, what's next?” Surprisingly, there also was some degree of confusion on how lung cancer screening was performed even among participants who had been screened for lung cancer. Although all in the screened group had had an LDCT, some of these participants reported that chest radiography was another method to screen for lung cancer.

#### Who should get screened for lung cancer

When asked who should get screened for lung cancer, most agreed that both current and former smokers should be screened. However, many participants described their perceptions that lung cancer screening is a ‘money‐making scam…like a bait and switch’. To provide context, many of those who had been screened had participated in screening in response to LDCTs being offered by many local health‐care systems (i.e. $49–$99). These participants referred to the low cost LDCT as a means to ‘get you in the door’, and then additional testing would be required if something abnormal was found. An illustrative comment follows and is reflective of a patient experience in a US health‐care system:

I hate to be kind of skeptical of modern technology…all of a sudden now we're hearing about lung screening. All of these years, how long have cigarettes been around, and how many people have died from lung cancer or whatever, now we have the screen. So they built the machine…it seems to me that some of these things that keeps coming up is almost like a scam. It's almost like a sales pitch…like telling you, come on, get this lung screening done and stuff like that, and just like everybody else here, the first time you go there, oh, we seen a spot, it was four millimeters, this and that, or whatever…come back in three months, come back in six months, bring your insurance card with you. Tell them to stop selling it.

#### Perceived benefits of lung cancer screening

Three perceived benefits of lung cancer screening were identified: (i) finding lung cancer early; (ii) giving peace of mind; and (iii) providing motivation to quit smoking. When asked about potential benefits of lung cancer screening, participants discussed finding lung cancer early as a benefit, noting that screening is likely associated with better survival rates. Comments such as ‘the earlier you catch it, the better possibility of a cure’ illustrate this perceived benefit of lung cancer screening. Some participants also pointed out that knowing what is ahead is beneficial. Participants discussed lung cancer screening as giving peace of mind if the results are negative, with comments such as ‘[knowing] everything is okay’ and receiving a ‘clean bill of health*’*. Finally, participants described being motivated to quit smoking as a potential unexpected benefit of lung cancer screening, noting it could serve as ‘a reminder that I need to work on my quitting*’*.

#### Perceived barriers to lung cancer screening

Three perceived barriers to lung cancer screening in general were described: (i) inconvenience; (ii) perceived smoking‐related stigma; and (iii) distrust of the health‐care system. When asked about potential barriers to lung cancer screening, participants described inconvenience as a barrier, particularly time constraints and scheduling conflicts. Participants consistently described perceived smoking‐related stigma as a potential barrier to lung cancer screening. Many participants described feeling smoking‐related stigma from younger health‐care providers, describing them as ‘people that don't know the culture we grew up in’. Most participants discussed stigma from the perspective of being blamed for having smoked, being made to feel like a social outcast and ‘making me feel like an idiot or stupid for smoking*’*. Finally, as described previously, participants reported distrust of government and the tobacco industry, and they reported uncertainty about the value of lung cancer screening, comparing ‘new machines to screen’ to a ‘scam*’*.

## Discussion

Several common themes were discovered among long‐term smokers regarding their perceptions about lung cancer, its associated risk factors and lung cancer screening. Although most individuals agreed that lung cancer is deadly, either confusion or inaccurate information existed as to the causes and associated risk factors. Participants seemed to assign greater importance to occupational and environmental exposure and placed less emphasis on smoking ‐ the number one risk factor for lung cancer that they all shared. These findings support and extend the work Faller *et al*. and Salander both finding that some smokers externalize the cause of lung cancer to the presence of toxins in the working environment or air pollution.^26^, ^27^ In addition, our study supports the findings of Weinstein in which some smokers attributed the cause of lung cancer to genetic pre‐disposition.^28^ Although acknowledging that occupational and environmental exposures and the potential synergistic effect of smoking with occupational and environmental factors are important, for many there seemed to be a disconnect or denial between risk factors for lung cancer and ways to prevent lung cancer. Although many participants identified tobacco smoking as a risk factor only late in the focus group discussion, these same participants quickly identified abstinence from smoking as a primary way to prevent lung cancer. This suggests that these current and former smokers were either not completely aware of or in denial of the critical role tobacco plays in lung cancer risk and aetiology. Increasing the awareness among long‐term smokers about the established links among lung cancer, tobacco and other aetiologies is important in order for long‐term smokers to accurately assess their risk for lung cancer.

Although participants reported that finding lung cancer early, giving peace of mind and being motivated to quit smoking were benefits of lung cancer screening, several important barriers to screening also were identified. One in particular, smoking‐related stigma, may serve as a barrier to lung cancer screening. Such stigma and its associated feelings of shame and self‐blame have been shown to influence timing of medical help‐seeking behaviour, and they are significantly associated with poorer quality of life, more depressive symptoms and lower levels of patient engagement in medical care.^29^, ^30^ In turn, less engagement in medical care is likely to be associated with decreased cancer screening participation.

Another important potential screening barrier among long‐term smokers may be related to distrust of the tobacco industry and government. Participants seemed to agree that the tobacco industry and government mutually worked for each other's interests and expressed a tremendous amount of scepticism and distrust about both systems which may be associated with health‐care system distrust. Heightened levels of distrust may impact the successful implementation of lung cancer screening programmes nationwide by serving as an impediment to screening behaviour. Careful attention to how lung cancer screening is marketed should include a focus on the shared decision‐making component in order to address issues of distrust. A helpful approach might be to involve screening‐eligible patients in the development of advertising materials and highlighting that lung cancer screening is the only screening modality to date in the US that requires a shared decision‐making visit for reimbursement.^8^ The shared decision‐making process is essential in helping an individual weigh the benefits and harms of screening in order to make an informed and mutually agreeable decision to screen or not screen. Responsible approaches to both the implementation and marketing of lung cancer screening can reinforce the importance of screening rather than unintentional perception that it is something to be feared.

It is also important to note that there were unscreened focus group participants who were either unaware or had misconceptions about lung cancer screening. Secondary to the new recommendation and expected, many did not know that lung cancer screening was available. However, several participants had misconceptions about how screening is performed. Screening is a concept that is commonly misconstrued; diagnostic testing in response to symptoms is frequently perceived as screening. As lung cancer screening is implemented, increased efforts to educate those at risk for lung cancer about the purpose of screening as a means of identifying potentially cancerous pulmonary nodules early will be key to success. In addition, because lung cancer screening involves a range of benefits and potential risks, it is essential that all screening‐eligible patients for whom lung cancer screening is recommended receive patient education and counselling. Further, integration of a shared decision‐making process is imperative and should include patient preference elicitation and values clarification prior to the patient's decision. Integration of this important process in lung cancer screening recommendations will help address the need to improve overall communication and decision making about screening.

## Limitations

As with all studies, results should be interpreted in the context of limitations. The greatest limitation in this study was lack of geographic sample diversity. Although the focus groups of unscreened participants were composed of individuals recruited from the community, the group of 12 screened participants was recruited from one health‐care system in one Midwestern city. Therefore, the results are potentially biased by the nuances of the local health‐care system. In addition, because 35% (*n* = 9) of the participants indicated a family history of lung cancer, this may have impacted their perceptions of lung cancer from personal experience. Future studies exploring lung cancer screening beliefs in screened individuals should include participants from multiple, geographically diverse health‐care systems to provide a more robust picture. However, one of the strengths of this study was demographic diversity. We had a diverse representation of ethnicity, ages and current and former smokers. However, the groups were not homogeneous in smoking status since each group included both current and former smokers, which may have influenced the dynamics of the discussions. Future studies should consider homogeneity of smoking status in constructing focus groups.

In addition, a specific focus on the risks of screening was not included and would likely have been helpful in the process of understanding perspectives on lung cancer screening. Future research should include an exploration of perceived risks and harms of screening. Finally, although every effort was made to encourage openness in the focus group discussions, the social pressure created by a focus group setting may have yielded different results than individual interviews. However, four separate focus groups were held and the themes that emerged were consistent across the different groups.

## Conclusion

Screening has the potential to identify lung cancer in long‐term smokers at an earlier stage, resulting in increased survival rates. Lung cancer screening is a new recommendation in the United States for long‐term smokers who meet age and pack‐year smoking parameters, but there are multilevel variables (i.e. individual, provider and health‐care system level) that will affect the successful implementation of such screening programmes. By understanding long‐term smokers' perceptions of lung cancer, its associated risk factors and lung cancer screening, we can address gaps in understanding for high risk, long‐term smokers in order to enhance the shared decision‐making process about this new screening modality. Most importantly, the scepticism that exists among lung cancer screening‐eligible patients makes a patient‐centred approach to lung cancer screening more critical. Health‐care providers must recognize and address both the benefits and potential risks of lung cancer screening and collaborate with their screening‐eligible patients towards a mutually agreeable shared decision that is right for both the individual's situation and preferences.

## Conflict of interests

The authors have no conflicts of interests to disclose.
